# Comparison of Community-Level and Patient-Level Social Risk Data in a Network of Community Health Centers

**DOI:** 10.1001/jamanetworkopen.2020.16852

**Published:** 2020-10-29

**Authors:** Erika K. Cottrell, Michelle Hendricks, Katie Dambrun, Stuart Cowburn, Matthew Pantell, Rachel Gold, Laura M. Gottlieb

**Affiliations:** 1 OCHIN Inc, Portland, Oregon; 2 Department of Family Medicine, Oregon Health and Science University, Portland; 3 Department of Pediatrics, University of California, San Francisco; 4 Kaiser Permanente Northwest Center for Health Research, Portland, Oregon; 5 Department of Family and Community Medicine, University of California, San Francisco

## Abstract

**Importance:**

Responding to the substantial research on the relationship between social risk factors and health, enthusiasm has grown around social risk screening in health care settings, and numerous US health systems are experimenting with social risk screening initiatives. In the absence of standard social risk screening recommendations, some health systems are exploring using publicly available community-level data to identify patients who live in the most vulnerable communities as a way to characterize patient social and economic contexts, identify patients with potential social risks, and/or to target social risk screening efforts.

**Objective:**

To explore the utility of community-level data for accurately identifying patients with social risks by comparing the social deprivation index score for the census tract where a patient lives with patient-level social risk screening data.

**Design, Setting, and Participants:**

Cross-sectional study using patient-level social risk screening data from the electronic health records of a national network of community health centers between June 24, 2016, and November 15, 2018, linked to geocoded community-level data from publicly available sources. Eligible patients were those with a recorded response to social risk screening questions about food, housing, and/or financial resource strain, and a valid address of sufficient quality for geocoding.

**Exposures:**

Social risk screening documented in the electronic health record.

**Main Outcomes and Measures:**

Community-level social risk was assessed using census tract–level social deprivation index score stratified by quartile. Patient-level social risks were identified using food insecurity, housing insecurity, and financial resource strain screening responses.

**Results:**

The final study sample included 36 578 patients from 13 US states; 22 113 (60.5%) received public insurance, 21 181 (57.9%) were female, 17 578 (48.1%) were White, and 10 918 (29.8%) were Black. Although 6516 (60.0%) of those with at least 1 social risk factor were in the most deprived quartile of census tracts, patients with social risk factors lived in all census tracts. Overall, the accuracy of the community-level data for identifying patients with and without social risks was 48.0%.

**Conclusions and Relevance:**

Although there is overlap, patient-level and community-level approaches for assessing patient social risks are not equivalent. Using community-level data to guide patient-level activities may mean that some patients who could benefit from targeted interventions or care adjustments would not be identified.

## Key Points

**Question:**

Can community-level data (eg, social deprivation index score of the census tract where patients live) accurately identify patient-level social risks?

**Findings:**

In this cross-sectional study including 36 578 patients, 10 858 (29.7%) screened positive for 1 or more social risks; 42% of patients with at least 1 social risk lived in neighborhoods not defined as disadvantaged.

**Meaning:**

Using community-level data to guide patient-level activities may result in missing some patients who can benefit from social risk–targeted or social risk–informed care.

## Teaser

This cross-sectional study explores the utility of community-level data for accurately identifying patients with social risks by comparing the social deprivation index score for the census tract where a patient lives with patient-level social risk screening data.

## Introduction

Responding to the substantial research on the association between social risk factors and health, enthusiasm has grown around social risk screening in health care settings, and numerous health systems in the US are experimenting with social risk screening initiatives.^1,2,3,4^ Recent studies have demonstrated the feasibility of implementing clinic-based screening and documenting social risks in electronic health records (EHRs),^5,6^ yet no clear standard has emerged on how to implement social risk screening, nor how clinicians can or should use social risk information to adjust patient care or make referrals to community resources.^6,7^ Moreover, some have questioned the benefit of integrating social risk screening into primary care, raising concerns about the additional burden of adding more required data collection to already busy primary care practices and the limited resources available to address identified social risk factors.^8,9,10^

In the absence of standard social risk screening recommendations, some health systems are exploring obtaining social risk information without screening patients directly.^11,12^ Community and neighborhood-level data characterizing the “conditions in which people are born, grow, live, work and age”^13^ are readily available from public sources, such as the US Census or American Community Survey, and can be geocoded and linked to patients’ addresses. Researchers have demonstrated the association between community-level measures of socioeconomic status and health.^14^ Theoretically, such data could provide an alternative way to identify patients with social risks or to target patients for whom self-reported screening should be prioritized.^11^ For example, community-level data could be used for “cold-spotting” to identify patients living in geographic areas that lack certain characteristics that support health, such as access to grocery stores and parks, higher rates of education and employment, clean air, and adequate housing.^15^ Identifying patients who live in the most vulnerable communities, or “cold spots,” could help clinics characterize and understand patients’ social and economic contexts and/or target social risk screening efforts.

However, relying solely on community-level data to understand the social context of an individual patient and/or to guide patient-level interventions poses a risk of ecological fallacy, or making erroneous assumptions about individuals based on aggregate information.^16,17^ Using patient-level social risk screening data from a national network of community health centers (CHCs),^18^ linked to geocoded data from the American Community Survey, we explored the utility of community-level data as a mechanism for identifying patients with social risks. Specifically, we compared the social deprivation index (SDI) score for the census tract where a patient lives with patient-level social risk screening data documented in the EHR to assess whether patients who live in “cold spots” have more patient-level social risks.^11,15^

## Methods

### Study Setting

For this cross-sectional study, we analyzed data from the OCHIN network of CHCs. OCHIN Inc is a nonprofit health center–controlled network that hosts a centrally managed instance of the Epic EHR (Epic Systems Corporation) for 645 CHC clinics across 21 US states. OCHIN CHCs provide care for the nation’s most vulnerable patients, the majority of whom are publicly insured or uninsured. Like most CHCs in the US, compared with the general population, patients who receive care at OCHIN CHCs are disproportionately poor, members of racial and ethnic minorities, and living with multiple chronic conditions. OCHIN hosts a research data warehouse that includes EHR data on more than 4.9 million patients, making it, to our knowledge, the largest single research-ready data source on US safety net patients. The research data warehouse also includes neighborhood and community-level data—also called community vital signs—from publicly available sources (eg, US Census, American Community Survey) that provide information about each patient’s community context. Patient addresses, collected by OCHIN network clinics, are geocoded to identify the census tract of each patient’s residence, then linked to the community vital signs data for that tract. The geocoding and linkage process and selection of measures are described in detail elsewhere.^12^

In 2016, OCHIN released a suite of EHR tools to help clinics document and review patient-reported social risk screening results.^19^ To meet the needs of OCHIN’s diverse members, these tools include several nationally recognized screening instruments (eg, PRAPARE, Centers for Medicare and Medicaid Services Accountable Health Communities, National Academy of Medicine [formerly Institute of Medicine] recommendations) encompassing 7 social risk domains: financial resource strain, food insecurity, housing instability, relationship safety, inadequate physical activity, social connection/isolation, and stress. In May 2017, a question was added asking patients if they would like help addressing any identified social risks. The CHCs have the option to choose an entire screening questionnaire (eg, PRAPARE) or individual social risk domains. This study followed the Strengthening the Reporting of Observational Studies in Epidemiology (STROBE) reporting guideline.

### Study Sample

This study was approved by the Western States Institutional Review Board. The study sample (N = 36 578) included patients screened for social risks in an OCHIN clinic between June 24, 2016, and November 15, 2018, who responded to at least 1 of the food, housing, or financial resource strain questions. A complete description of the exclusion criteria is provided in the Figure. This study uses social risk screening data collected during clinical care and documented in the EHR. As reported elsewhere, CHCs use a variety of different criteria in selecting patients for social risk screening, and there are significant differences between patients who are screened and not screened.^5,18^ Patients were excluded if they did not have a residential address in the same state as where the screening occurred (to enable meaningful comparison of screening responses and census-tract level information) or if they lacked an address of adequate quality to enable geocoding. Patients who recently moved and/or those who traveled across state lines to receive care may have had a higher likelihood of being excluded. Finally, patient address data were documented by the individual CHC, and we were unable to determine what factors influenced address quality. This could have introduced bias if address data were not missing at random.

**Figure.**
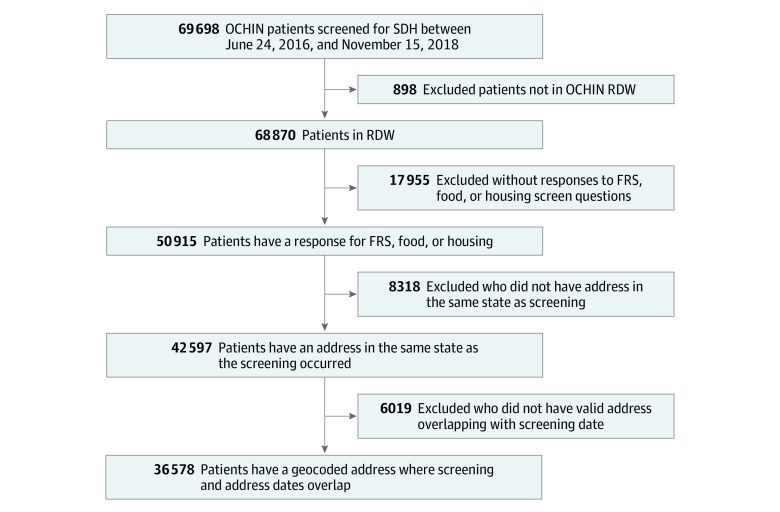
Derivation of Study Sample FRS indicates financial resource strain; RDW, research data warehouse; SDH, social determinants of health.

### Measures

#### Community-Level Social Risk

To quantify community-level social risk, all patients with a valid address in the OCHIN research data warehouse were assigned a census-tract level SDI score using information on their last available address recorded in the EHR. Originally developed by Butler et al^20^ and updated in 2015,^21^ the SDI is a composite measure of 7 demographic characteristics from the American Community Survey, including percentage living in poverty, percentage with less than 12 years of education, percentage of single-parent households, percentage living in a rented housing unit, percentage living in an overcrowded housing unit, percentage of households without a car, and percentage of nonemployed adults younger than 65 years. Census tracts with higher percentile scores have higher levels of social deprivation relative to other census tracts nationwide. For example, a census tract with an SDI score of 75 is considered “worse off” than 75% of census tracts in the US. On the other hand, a census tract with an SDI score of 5 is considered “better off” than 95% of census tracts in the US. This multidimensional measure has been shown to be more strongly associated with health outcomes than poverty alone.^18^ Moreover, prior studies suggest that patients living in cold spots—defined as those census tracts with an SDI score in the highest quartile nationally (≥75)—have worse health outcomes relative to those in more resource-rich tracts.^11^

#### Patient-Level Social Risk

Patient-level measures of food insecurity, housing insecurity, and financial resource strain were included in our analysis. We classified patients as having social risks if they screened positive for 1 or more of these domains (see eTable 1 in the Supplement for a description of screening questions for each of these domains). These 3 domains were selected for several reasons. First, given the lack of standardized screening recommendations, CHCs are implementing screening in a variety of ways. Instead of using an established tool (eg, PRAPARE, Accountable Health Communities), many CHCs have opted to focus on screening for specific social risk domains. To date, food insecurity, housing insecurity, and financial resource strain are among the most frequently documented social risk domains in OCHIN CHCs, in part because they are actionable (eg, through referrals to local resources).^18^ Second, relative to other patient-level measures that focus on psychosocial domains (eg, relationship safety, social connection/isolation, stress), these domains are most aligned with the socioeconomic factors integrated into the SDI. Third, despite the limited consensus on standardized SDH screening recommendations, there is emerging consensus around standardized questions to assess food insecurity, housing insecurity, and financial resource strain.

OCHIN’s EHR-based screening tools include standardized question and response categories for each of these domains. Food insecurity is assessed using a validated 2-item screen called the Hunger Vital Sign.^22^ Housing insecurity is assessed differently depending on when the patient received the screening tool. Before May 17, 2018, patients were asked 2 items from the HealthBegins Upstream Risk screening tool about their living situation in the past month.^23^ This has since transitioned to a 2-item question from the Accountable Health Communities screening tool about stability and quality of their housing.^2^ Financial resource strain was assessed using the National Academy of Medicine–recommended screening question.^5,19,24^ Finally, based on user feedback in pilot CHCs, screening questions were added in May 2017 asking whether patients desired CHC assistance in addressing identified risk factors.^19^ When available, data on whether patients wanted help addressing their social risks were also extracted (see eTable 1 in the Supplement for a list of measures and response options).

### Statistical Analysis

Descriptive statistics (counts and proportions) were generated to characterize the study sample. First, each patient in the study sample was categorized into national quartiles based on the SDI of their census tract of residence (Q1: 1 to <25; Q2: 25 to <50; Q3: 50 to <75; Q4: 75-100). Second, the numbers and percentages of patients who reported food insecurity, housing insecurity, or financial resource strain were calculated, overall and within each SDI quartile. We then compared the percentage of patients who screened positive for 1 or more social risk factors in each SDI quartile as well as between cold spots (Q4 census tracts) and non–cold spots (Q1-Q3 census tracts). Given the exploratory nature of this study, no hypothesis testing was performed. Data analysis was performed between January 2019 and July 2019.

## Results

### Sample Characteristics

Of the 36 578 patients included in the final study sample, 60.5% (n = 22 113) received public insurance, 57.9% (n = 21 181) were female, 19.2% (n = 7013) were 19 years or younger at the time of screening, and 20.3% (n = 7422) were 60 years or older (Table 1). The majority of study patients were White (n = 17 578; 48.1%), with 29.8% (10 918) identifying as Black/African American, and 23.0% (8431) as Hispanic. A small percentage (n = 1725; 4.7%) were recorded as homeless, and 30% spoke a primary language other than English. Screening occurred at 275 clinics (43% of all OCHIN clinics) across 13 states, with the majority residing in Massachusetts (55.7%), Oregon (18.5%), or California (12.4%) (see eTable 2 in the Supplement).

**Table 1.** Demographic Characteristics of the Sample^a^

Characteristic	No. (%) (N = 36 578)
Age, y	
0-9	3155 (8.6)
10-19	3858 (10.5)
20-29	5114 (14.0)
30-39	5453 (14.9)
40-49	5180 (14.2)
50-59	6214 (17.0)
60-69	4521 (12.4)
≥70	2901 (7.9)
Missing or unknown	182 (0.5)
Sex	
Female	21 181 (57.9)
Male	15 396 (42.1)
Missing or unknown	1 (0)
Race	
American Indian/Alaska Native	186 (0.5)
Asian	3081 (8.4)
Black/African American	10 918 (29.8)
Native Hawaiian/Pacific Islander	443 (1.2)
White	17 578 (48.1)
Multiple races	215 (0.6)
Missing or unknown	4157 (11.4)
Ethnicity	
Hispanic	8431 (23.0)
Non-Hispanic	25 290 (69.1)
Missing or unknown	2857 (7.8)
Preferred language	
English	25 289 (69.1)
Spanish	5723 (15.6)
Other	5160 (14.1)
Missing or unknown	406 (1.1)
Homeless status	
Homeless	1725 (4.7)
Not homeless	31 384 (85.8)
Missing or unknown	3469 (9.5)
Current insurance status	
Medicaid	16 129 (44.1)
Medicare	4607 (12.6)
Other public	1377 (3.8)
Private	8866 (24.2)
Uninsured	2505 (6.8)
Missing or unknown	3094 (8.5)
State of residence	
California	4549 (12.4)
Georgia	1022 (2.8)
Indiana	567 (1.6)
Massachusetts	20 366 (55.7)
Minnesota	739 (2.0)
Montana	17 (0)
North Carolina	847 (2.3)
New Mexico	2 (0)
Ohio	332 (0.9)
Oregon	6753 (18.5)
Texas	40 (0.1)
Washington	1076 (2.9)
Wisconsin	268 (0.7)
Census tract SDI score, national quartile	
Q1 (1-<25)	1869 (5.1)
Q2 (25-<50)	4926 (13.5)
Q3 (50-<75)	8585 (23.5)
Q4 (75-100)	21 197 (58.0)

Abbreviation: SDI, social deprivation index.

^a^ Patient demographic characteristics as of the date of their first screening encounter between June 24, 2016, and November 15, 2018.

A total of 58.0% of patients in the study sample (n = 21 197) lived in cold spot census tracts with an SDI score in the highest quartile (≥75 or Q4; most deprived), and 42.0% lived in non–cold spot census tracts (23.5% [n = 8585] lived in a Q3 census tract; 13.5% [n = 4926] in a Q2 census tract; and 5.1% [n = 1869] in a Q1 census tract) (Table 1). In total, 88.4% of sample patients (n = 32 337) had documented responses to questions about housing insecurity (n = 3880; 12.0% screened positive), 66.8% (n = 24 426) had documented responses to questions about food insecurity (n = 6839; 28.0% screened positive), and 39.0% (14 276) had documented responses to questions about financial resource strain (7145; 50.0% screened positive) (Table 2). Because CHC programs and incentives differ regionally, patterns of screening differed by state (see eTable 2 in the Supplement).

**Table 2.** Social Risk Screening Domains and Responses

Social risk screening domains^a^	Patients with documented response (N = 36 578), No. (% of sample)	Screen, No. (%)	
		Positive	Negative
Financial resource strain	14 276 (39.0)	7145 (50.0)	7131 (50.0)
Food insecurity	24 426 (66.8)	6839 (28.0)	17 587 (72.0)
Housing insecurity	32 337 (88.4)	3880 (12.0)	28 457 (88.0)

^a^ See eTable 1 in the Supplement for social risk screening questions and coding of positive and negative screening responses.

### Census Tract SDI Quartile and Patient-Reported Social Risks

Overall, approximately 29.7% of sample patients (n = 10 858) screened positive for housing insecurity, food insecurity, and/or financial resource strain (Table 3). Of these, 60.0% (n = 6516) resided in a Q4 census tract, 22.9% in Q3 (n = 2491), 13.0% in Q2 (n = 1415), and 4.0% (n = 436) in Q1. The percentage of patients who screened positive within each quartile was relatively stable across Q2 to Q4, with 6516 of 21 197 patients (30.7%) screening positive in the highest SDI quartile (Q4 or cold spot), 2491 of 8585 (29.0%) in the third quartile, and 1415 of 4926 (28.7%) in the second quartile. Although the first-quartile SDI had the fewest number of patients screened (1869), almost a quarter (436, 23.3%) screened positive for at least 1 risk factor (Table 3).

**Table 3.** Social Risk Screening Responses by Census Tract Social Deprivation (SDI) Score (National Quartiles)

Social risk screening response	Census tract-level SDI quartile, No. (column %, row %)				Total, No. (%)
	Q1 (1-<25)	Q2 (25-<50)	Q3 (50-<75)	Q4 (75-100)	
≥1 Social risk factor (positive screening)^a^	436 (23.3, 4.0)	1415 (28.7, 13.0)	2491 (29.0, 22.9)	6516 (30.7, 60.0)	10 858 (30.0)
No social risk factors (negative screening)	1433 (76.7, 5.6)	3511 (71.3, 13.7)	6094 (71.0, 23.7)	14 681 (69.3, 57.1)	25 719 (70.0)
Total, No.	1869	4926	8585	21 197	36 578
Yes, I need help addressing social risks^b^	46 (48.0, 5.1)	113 (29.5, 12.4)	174 (37.7, 19.2)	575 (35.5, 63.3)	908 (35.5)
No, I do not need help addressing social risks^b^	50 (52.1, 3.0)	270 (70.5, 16.3)	287 (62.3, 17.4)	1046 (64.5, 63.3)	1653 (64.5)
Total, No.	96	383	461	1621	2561

^a^ Patients who screened positive in at least 1 domain (financial resource strain, food insecurity, and/or housing insecurity).

^b^ Patients who screened positive were asked if they would like assistance addressing identified social risk factors.

Of the patients who screened positive for 1 or more social risks (n = 10 858), 23.6% (n = 2561) were asked whether they wanted help from clinic staff to address identified risks. Of those, 35.5% (n = 908) said they wanted help. Overall, 63.3% (n = 575) of those who wanted help lived in a cold spot census tract (Q4), with 19.2% (n = 174) in Q3; 12.4% (n = 113) in Q2; and 5.1% (n = 46) in Q1 (Table 3). Interestingly, when looking at the percentage who wanted help within each quartile, Q1 had the highest percentage of patients who wanted help to address identified risks (n = 46; 48.0%). Conversely, Q4 had the highest percentage of patients who said they did not want help (n = 1046; 64.5%) (Table 3).

Of those who screened positive for at least 1 social risk factor, 60.0% (n = 6516) resided in a cold spot census tract and would have been correctly identified as having a risk using a cold-spotting approach. However, 40.0% (n = 4342) of patients reporting 1 or more social risks would not be correctly identified (Table 4). Of the 25 719 patients who did not screen positive for any social risk, 57.1% (n = 14 681) resided in a cold spot and thus would be incorrectly identified as having social risk using a cold-spotting approach (Table 4). Overall, the accuracy of the community-level data for identifying patients with and without social risks was 48.0% (n = 17 544).

**Table 4.** Social Risk Screening Response by Cold Spot Status

Social risk screening response	No. (column %, row %)		Total, No.
	Cold spot^a^	Non–cold spot^b^	
≥1 Social risk factor (positive screening)^c^	6516 (30.7, 60.0)	4342 (28.2, 40.0)	10 858
No social risk factors (negative screening)	14 681 (69.3, 57.1)	11 038 (71.8, 42.9)	25 719
Total, No.	21 197	15 380	36 577

^a^ Census-tract level social deprivation index (SDI) percentile score 75-100 (quartile 4).

^b^ Census-tract level SDI percentile score <75 (quartiles 1-3).

^c^ Patients who screened positive in at least 1 domain (financial resource strain, food insecurity, and/or housing insecurity).

## Discussion

There is growing recognition of the integral role of the health care sector in identifying and addressing social factors and the importance of considering social context to improve health equity.^2,3,4,7,25^ Yet critical knowledge gaps remain, and there is a limited evidence base regarding best practices for integrating social and medical care.^7^ In the midst of this environment, numerous approaches have emerged, including both patient-level screening and efforts that leverage community-level social risk data, sometimes in combination.^1,5,6,10,18,26,27,28,29,30,31^ Recognizing the challenge, cost, and time involved with implementing patient-level social risk screening initiatives, some health care systems are exploring strategies for using publicly available community-level data as proxies of patient-level social and economic information and/or as a way to target social risk–targeted (directly addressing social risk factors) or social risk–informed care (adapting care to accommodate social risks).^6,7,8,9,10,27,32^ In this study, we assessed concordance between patient-reported social risks and community-level social deprivation among patients receiving care in a national network of CHCs. Building on previous work,^10,11^ we defined cold spots as census tracts with SDI scores within the highest quartile nationally. Although there was some overlap between cold spot status and the presence of patient-level social risks, with 60.0% of those who reported at least 1 social need living in a cold spot census tract, 40.0% of patients who screened positive for at least 1 social risk did not live in a cold spot. Overall, the accuracy of the community-level data for identifying patients with and without social risks was 48.0%.

These findings have several implications for the use of patient-level and community-level social risk data in clinical settings. First, using community-level data as a proxy for patient-level social risk screening or to refine targets for patient-level data collection^10,29^ may heighten the risk of ecologic fallacy, wherein incorrect assumptions are made about an individual based on aggregate-level information about a group.^6,16^ Despite the potential utility of community-level data for a variety of purposes, including in cases when universal screening is not feasible, findings within our study population suggest that ecologic fallacy may in fact be an issue when using community-level data to identify patient-level social needs. Indeed, based on data from the OCHIN network, if a CHC were to use the cold spot approach to identify patients for screening or to implement a social care intervention, they would risk missing 40.0% (n = 4342) of patients with social risks who live in more affluent areas and/or incorrectly target 57.1% (n = 14 681) of patients without social risks who live in a cold spot.

Second, our results suggest that, when asked, a larger percentage of patients said that they did not want help addressing identified risks (64.5% [n = 1653] said that they did not want help vs 35.5% [n = 908] who did). This finding is supported by a 2019 qualitative study,^33^ which found that although patients and caregivers believed social risk screening was important and acceptable, they did not expect their health care teams to address the social challenges they faced. Moreover, despite the low numbers, the counterintuitive finding that a higher percentage of patients within the most affluent quartile (Q1) said they wanted help could be indicative of a greater availability of resources in these areas, but this is unclear. Overall, these findings underscore the need for additional research to explore patient perspectives on social care screening and referrals, including whether and how health care teams should address identified risk factors. They also raise questions about whether and how the availability of resources in a patient’s neighborhood or community—either perceived or actual—might influence their desire for help.

Third, our findings underscore the importance of identifying and exploring the potential for other less intensive strategies for understanding patient-level social risks. Medical professionals, especially those in primary care, have articulated the range of challenges involved in identifying and addressing social risks, including the increased burden of integrating these activities into an already burgeoning workoad.^9,10,11,12^ One strategy would be to lower the burden of patient-level screening. There are several multiquestion screening instruments,^2,3,4^ variability in measures, and limited understanding of their psychometric properties (ie, reliability and validity).^34^ Potential next steps could include identifying a smaller set of risk factors to screen for in clinical settings, or alternatively, developing a valid and reliable single-question screening for social risks.

Finally, although our findings suggest that census tract-level SDI is not always accurate in identifying patient-level social risks, community-level data still have enormous value if targeted appropriately. As others have articulated, community-level social determinants of health or “community vital signs”—such as the availability of green spaces for walking, or environmental factors that might affect health—provide important contextual information about a patient’s environment that could inform care.^12,35^ Beyond their utility in contextualizing patient care, community-level data are a vital source of information for community-level interventions (eg, advocacy, alignment) and could be used to inform the development value-based payment structures or approaches to risk adjustment.^6^ Indeed, other countries have demonstrated the value of using area-based measures of socioeconomic variation to assess community needs, inform research, adjust clinical funding, allocate community resources, and determine policy impact.^36^ More research is needed to understand how patient-level and community-level data can be used in concert to most effectively and efficiently invest limited resources.

### Limitations

This study has several limitations. First, a primary limitation of our findings is the generalizability of the study sample. Patients who completed social risk assessments were a nonrandom sample of OCHIN patients and represented less than 1% of OCHIN patients seen during the study period. There was significant variation among OCHIN CHCs in the number of screenings conducted, the types of patients selected for screening, and the social risk questions that were asked.^18^ Because of the limitations of these EHR data, we cannot determine if differing response rates across SDH domains can be attributed to patient refusal to answer or clinics’ decisions not to ask them. Bias resulting from missing, inaccurate, or inadequate address data are also unknown. Second, this was an exploratory study using data from a relatively limited period and at the very beginning of social risk screening implementation in CHCs. Preliminary estimates from OCHIN CHCs suggest that the number of documented screenings is continuing to increase steadily. Future research should seek to replicate these results using a larger sample of patients and over an extended time frame. Third, we were unable to ascertain whether the SDI performed better in different geographic regions and/or in urban vs rural areas. Future research should explore whether the risk of misclassification varies between these geographic classifications and/or whether the SDI has more utility in some circumstances than in others. Finally, there are numerous community-level measures, beyond the SDI, that are available for public use. Although our findings suggest that relying on SDI in place of patient-level social risk screening may not be sufficient, future research should explore the utility of other measures of community-level social context for identifying high-risk individuals.

## Conclusions

Our findings suggest that patient-level and community-level approaches to incorporating social risk data in clinical contexts are not equivalent. Although there is overlap in patients identified using each method, 48.0% of patients are misclassified, and many patients in less disadvantaged neighborhoods desired assistance. Using community-level social risk data to guide patient-level activities may mean that some patients who could benefit from programs targeting social conditions or care adjustments would not be identified.

## Notes

Supplement. **eTable 1.** Social Risk Measures and Response Options **eTable 2.** Social Risk Screening Domains and Total Patients Screened by State

## References

[1] Cottrell EK, Gold R, Likumahuwa S, Using health information technology to bring social determinants of health into primary care: a conceptual framework to guide research. J Health Care Poor Underserved. 2018;29(3):949-963. doi:10.1353/hpu.2018.0071 30122675 PMC6779030

[2] National Association of Community Health Centers Protocol for Responding to and Assessing Patients’ Assets, Risks, and Experiences (PRAPARE). Accessed September 28, 2020. http://www.nachc.org/research-and-data/prapare/

[3] Institute of Medicine Committee on the Recommended Social and Behavioral Domains and Measures for Electronic Health Records Capturing Social and Behavioral Domains and Measures in Electronic Health Records: Phase 2. National Academies Press; 2014. 24757748

[4] Alley DE, Asomugha CN, Conway PH, Sanghavi DM Accountable health communities—addressing social needs through Medicare and Medicaid. N Engl J Med. 2016;374(1):8-11. doi:10.1056/NEJMp1512532 26731305

[5] Gold R, Bunce A, Cowburn S, Adoption of social determinants of health EHR tools by community health centers. Ann Fam Med. 2018;16(5):399-407. doi:10.1370/afm.2275 30201636 PMC6131002

[6] Gottlieb LM, Francis DE, Beck AF Uses and misuses of patient- and neighborhood-level social determinants of health data. Perm J. 2018;22:18-078. doi:10.7812/TPP/18-078 30227912 PMC6141653

[7] National Academies of Sciences, Engineering, and Medicine Integrating Social Care Into the Delivery of Health Care: Moving Upstream to Improve the Nation’s Health. National Academies Press; 2019. 31940159

[8] Kaufman A Theory vs practice: should primary care practice take on social determinants of health now? yes. Ann Fam Med. 2016;14(2):100-101. doi:10.1370/afm.1915 26951582 PMC4781510

[9] Solberg LI Theory vs practice: should primary care practice take on social determinants of health now? no. Ann Fam Med. 2016;14(2):102-103. doi:10.1370/afm.1918 26951583 PMC4781511

[10] Tong ST, Liaw WR, Kashiri PL, Clinician experiences with screening for social needs in primary care. J Am Board Fam Med. 2018;31(3):351-363. doi:10.3122/jabfm.2018.03.170419 29743219 PMC6497466

[11] Liaw W, Krist AH, Tong ST, Living in “cold spot” communities is associated with poor health and health quality. J Am Board Fam Med. 2018;31(3):342-350. doi:10.3122/jabfm.2018.03.170421 29743218 PMC7085304

[12] Bazemore AW, Cottrell EK, Gold R, “Community vital signs”: incorporating geocoded social determinants into electronic records to promote patient and population health. J Am Med Inform Assoc. 2016;23(2):407-412. doi:10.1093/jamia/ocv088 26174867 PMC11740537

[13] World Health Organization Social determinants of health. Accessed December 15, 2019. https://www.who.int/social_determinants/sdh_definition/en/

[14] Krieger N, Chen JT, Waterman PD, Rehkopf DH, Subramanian SV Race/ethnicity, gender, and monitoring socioeconomic gradients in health: a comparison of area-based socioeconomic measures—the public health disparities geocoding project. Am J Public Health. 2003;93(10):1655-1671. doi:10.2105/AJPH.93.10.1655 14534218 PMC1448030

[15] Westfall JM Cold-spotting: linking primary care and public health to create communities of solution. J Am Board Fam Med. 2013;26(3):239-240. doi:10.3122/jabfm.2013.03.130094 23657689

[16] Robinson WS Ecological correlations and the behavior of individuals. Am Sociol Rev. 1950;15(3):351-357. doi:10.2307/2087176 19179346

[17] Diez-Roux AV Bringing context back into epidemiology: variables and fallacies in multilevel analysis. Am J Public Health. 1998;88(2):216-222. doi:10.2105/AJPH.88.2.216 9491010 PMC1508189

[18] Cottrell E, Dambrun K, Cowburn S, Variation in electronic health record documentation of social determinants of health across a national network of community health centers. Am J Prev Med. 2019;57(6)(suppl 1):S65-S73. doi:10.1016/j.amepre.2019.07.014 31753281

[19] Gold R, Cottrell E, Bunce A, Developing electronic health record (EHR) strategies related to health center patients’ social determinants of health. J Am Board Fam Med. 2017;30(4):428-447. doi:10.3122/jabfm.2017.04.170046 28720625 PMC5618800

[20] Butler DC, Petterson S, Phillips RL, Bazemore AW Measures of social deprivation that predict health care access and need within a rational area of primary care service delivery. Health Serv Res. 2013;48(2 pt 1):539-559. doi:10.1111/j.1475-6773.2012.01449.x 22816561 PMC3626349

[21] Robert Graham Center Social deprivation index (SDI). Accessed September 28, 2020. https://www.graham-center.org/rgc/maps-data-tools/sdi/social-deprivation-index.html

[22] Hager ER, Quigg AM, Black MM, Development and validity of a 2-item screen to identify families at risk for food insecurity. Pediatrics. 2010;126(1):e26-e32. doi:10.1542/peds.2009-3146 20595453

[23] Manchanda R, Gottlieb L Upstream Risks Screening Tool and Guide Version 2.6. HealthBegins; 2015.

[24] Puterman E, Adler N, Matthews KA, Epel E Financial strain and impaired fasting glucose: the moderating role of physical activity in the Coronary Artery Risk Development in Young Adults study. Psychosom Med. 2012;74(2):187-192. doi:10.1097/PSY.0b013e3182448d74 22286855

[25] Adler NE, Stead WW Patients in context—EHR capture of social and behavioral determinants of health. N Engl J Med. 2015;372(8):698-701. doi:10.1056/NEJMp1413945 25693009

[26] Gottlieb L, Sandel M, Adler NE Collecting and applying data on social determinants of health in health care settings. JAMA Intern Med. 2013;173(11):1017-1020. doi:10.1001/jamainternmed.2013.560 23699778

[27] Gottlieb L, Tobey R, Cantor J, Hessler D, Adler NE Integrating social and medical data to improve population health: opportunities and barriers. Health Aff (Millwood). 2016;35(11):2116-2123. doi:10.1377/hlthaff.2016.0723 27834254

[28] Beck AF, Klein MD, Kahn RS Identifying social risk via a clinical social history embedded in the electronic health record. Clin Pediatr (Phila). 2012;51(10):972-977. doi:10.1177/0009922812441663 22511197

[29] Beck AF, Sandel MT, Ryan PH, Kahn RS Mapping neighborhood health geomarkers to clinical care decisions to promote equity in child health. Health Aff (Millwood). 2017;36(6):999-1005. doi:10.1377/hlthaff.2016.1425 28583957 PMC5813285

[30] Byhoff E, Cohen AJ, Hamati MC, Tatko J, Davis MM, Tipirneni R Screening for social determinants of health in Michigan health centers. J Am Board Fam Med. 2017;30(4):418-427. doi:10.3122/jabfm.2017.04.170079 28720624

[31] DeVoe JE, Bazemore AW, Cottrell EK, Perspectives in primary care: a conceptual framework and path for integrating social determinants of health into primary care practice. Ann Fam Med. 2016;14(2):104-108. doi:10.1370/afm.1903 26951584 PMC4781512

[32] Hessler D, Bowyer V, Gold R, Shields-Zeeman L, Cottrell E, Gottlieb LM Bringing social context into diabetes care: intervening on social risks versus providing contextualized care. Curr Diab Rep. 2019;19(6):30. doi:10.1007/s11892-019-1149-y 31037356

[33] Byhoff E, De Marchis EH, Hessler D, Part II: a qualitative study of social risk screening acceptability in patients and caregivers. Am J Prev Med. 2019;57(6)(suppl 1):S38-S46. doi:10.1016/j.amepre.2019.07.016 31753278 PMC6876708

[34] Henrikson NB, Blasi PR, Dorsey CN, Psychometric and pragmatic properties of social risk screening tools: a systematic review. Am J Prev Med. 2019;57(6)(suppl 1):S13-S24. doi:10.1016/j.amepre.2019.07.012 31753276

[35] Bazemore A, Phillips RL, Miyoshi T Harnessing geographic information systems (GIS) to enable community-oriented primary care. J Am Board Fam Med. 2010;23(1):22-31. doi:10.3122/jabfm.2010.01.090097 20051539

[36] Phillips RL, Liaw W, Crampton P, How other countries use deprivation indices—and why the United States desperately needs one. Health Aff (Millwood). 2016;35(11):1991-1998. doi:10.1377/hlthaff.2016.0709 27834238

