# Supplementary material for: Comparison of Community-Level and Patient-Level Social Risk Data in a Network of Community Health Centers
Source: JAMA Netw Open. 2020 Oct 29;3(10):e2016852. doi: 10.1001/jamanetworkopen.2020.16852 (PMC7596576; doi:10.1001/jamanetworkopen.2020.16852)
Supplement: Supplement. — eTable 1. Social Risk Measures and Response Options eTable 2. Social Risk Screening Domains and Total Patients Screened by State [file jamanetwopen-e2016852-s001.pdf]

Supplemental Online Content

Cottrell EK, Hendricks M, Dambrun K, et al. Comparison of community-level and patient-level social risk data in a network of community health centers. *JAMA Netw Open.* 2020;3(10):e2016852. doi:10.1001/jamanetworkopen.2020.16852

eTable 1. Social Risk Measures and Response Options

eTable 2. Social Risk Screening Domains and Total Patients Screened by State

This supplemental material has been provided by the authors to give readers additional information about their work.

© 2020 Cottrell et al. *JAMA Network Open.*

eTable 1. Social Risk Measures and Response Options

Domain	Measure	Response
Housing Insecurity	In the last month: a) have you slept outside, in a shelter, or in a place not meant for sleeping?; or b) have you had any concerns about the conditions and quality of your housing?	**Yes;** No; I choose not to answer
	After May 17, 2018<br>What is your living situation today?	I have a steady place to live; **I have a place to live today, but am worried about losing it in the future; I do not have a steady place to live;** I choose not to answer
	Do have any problems with the following?	**Pests; Mold; Lead paint or pipes; Lack of heat; Oven or stove not working; Smoke detectors missing or not working; Water leaks;** None of the above; I choose not to answer
Food Insecurity	In the past 12 months, a) I/we worried that my/our food would run out before I/we got money to buy more; or b) the food I/we bought didn't last and I/we did not have the money to buy more.	**Often true; Sometimes true;** Never true; I choose not to answer
Financial Resource Strain	How hard is it to pay for the basics, such as food, housing, heating, medical care and medications?	Not at all; **Somewhat hard; Very hard;** I choose not to answer
Do you want help?	Would you like assistance with any of the above items?	**Yes**; No

Bolded responses=**Social risk factor (patients who select any of the bolded responses would be identified as screening positive for that domain)**

© 2020 Cottrell et al. *JAMA Network Open.*

eTable 2. Social Risk Screening Domains and Total Patients Screened by State

State of Residence	Social Risk Screening Domain			Total Patients Screened N (column %)
	Financial Resource Strain N (row %)	Food Insecurity N (row %)	Housing Insecurity N (row %)	
California	4368 (96)	4513 (99)	4516 (99)	4,549 (12.4)
Georgia	988 (96)	997 (98)	1003 (98)	1,022 (2.8)
Indiana	557 (98)	560 (99)	562 (99)	567 (1.6)
Massachusetts	1197 (6)	8959 (44)	18974 (93)	20,366 (55.7)
Minnesota	729 (99)	736 (99)	739 (100)	739 (2.0)
Montana	14 (82)	17 (100)	17 (100)	17 (0.1)
North Carolina	812 (96)	825 (97)	832 (98)	847 (2.3)
New Mexico	2 (100)	1 (50)	2 (100)	2 (.01)
Ohio	308 (93)	313 (94)	321 (97)	332 (0.9)
Oregon	4247 (63)	6220 (92)	4230 (63)	6,753 (18.5)
Texas	38 (95)	40 (100)	40 (100)	40 (0.1)
Washington	1004 (93)	991 (92)	1068 (99)	1,076 (2.9)
Wisconsin	12 (5)	255 (95)	34 (13)	268 (0.7)
Total	14,276	24,427	32,338	36,578

© 2020 Cottrell et al. *JAMA Network Open.*
